# Symptomatic Hypoalbuminemia as an Indication for Treatment of Waldenström Macroglobulinemia: A Case Report and Review of the Literature

**DOI:** 10.1155/2019/1929124

**Published:** 2019-10-03

**Authors:** Jeremy T. Larsen, Frederick D. Leonard

**Affiliations:** ^1^Division of Hematology and Medical Oncology, Mayo Clinic, Phoenix, Arizona, USA; ^2^International Waldenstrom's Macroglobulinemia Foundation (IWMF), Sarasota, Florida, USA

## Abstract

Hypoalbuminemia has been reported as common in patients with symptomatic Waldenström macroglobulinemia (WM), but it is not typically mentioned as a manifestation of the disease in review articles or articles discussing indications for treatment. We present the case of a patient with WM for whom symptomatic hypoalbuminemia was the primary reason for initiating treatment. Except in rare cases of WM with renal or gastrointestinal involvement, hypoalbuminemia is thought to be due to the effects of elevated levels of inflammation-associated cytokines, and it has been associated with greater likelihood of progression of asymptomatic to symptomatic disease, greater disease severity, and poorer prognosis. Hypoalbuminemia in WM may become symptomatic, and it may be a reason to initiate treatment if symptoms affect quality of life.

## 1. Introduction

Waldenström macroglobulinemia (WM) is a B-cell lymphoproliferative disorder characterized by bone marrow infiltration with lymphoplasmacytic cells that secrete monoclonal immunoglobulin M (IgM). Its clinical manifestations can involve virtually any organ system since disease effects are due to both cellular infiltration and circulating IgM.

There is currently no cure for WM, so treatment is initiated when the disease becomes life-threatening or symptoms significantly affect quality of life. Suggested indications for treatment include symptomatic anemia with hemoglobin less than 10 g/dL; thrombocytopenia less than 100 × 10^9^/L; symptomatic lymphadenopathy or hepatosplenomegaly; symptomatic hyperviscosity; symptomatic cryoglobulinemia, cold agglutinins, autoimmune-related events, or amyloidosis; moderate to severe peripheral neuropathy; constitutional symptoms (high fever, drenching sweats, significant weight loss, and severe fatigue); and symptomatic central nervous system involvement (Bing–Neel syndrome) [1, 2].

Not mentioned in the literature as an indication for treatment is symptomatic hypoalbuminemia. We present the case of a patient with WM for whom this was the primary reason for initiating treatment.

## 2. Case Presentation

A 66-year-old physician saw his internist for a routine annual visit. Over the previous six months, he had noted some decreased stamina while riding his mountain bike, but he was otherwise without symptoms. His exam was unremarkable, but initial laboratory studies showed a hemoglobin of 12.5 g/dL (reference value 13.2–16.6 g/dL) and an elevated total protein. Additional testing demonstrated an M spike of 2.4 g/dL. Albumin was low-normal at 3.5 g/dL (reference value 3.5–5.0 g/dL). A year prior, his hemoglobin and albumin had been within the normal range at 14.2 g/dL and 3.9 g/dL, respectively. In retrospect, these were both trending downward compared to previous results, and his hemoglobin had historically been significantly higher (15-16 g/dL) since he was quite physically fit and lived at 5,000 ft. elevation.

He was referred to our institution for further evaluation. A bone marrow biopsy, including cytogenetics, fluorescence in situ hybridization (FISH), and flow cytometry, was consistent with a diagnosis of WM with 70% bone marrow involvement by lymphoplasmacytic cells. Serum protein electrophoresis and immunofixation demonstrated an M spike of 2.1 g/dL with IgM kappa monoclonal protein. Quantitative IgM was 3,760 mg/dL (reference value 37–286 mg/dL), and hemoglobin was 12.7 g/dL. C-reactive protein (CRP) was 14.7 mg/L (reference range ≤8.0 mg/L). Lactate dehydrogenase and a comprehensive metabolic panel were normal with the exception of elevated total protein and slightly elevated alkaline phosphatase. Albumin was 3.7 g/dL. Twenty-four-hour urine protein was 269 mg (reference value <229 mg/24 h) of which 14% was albumin. Also present was an M spike of 111 mg/24 h. Follow-up a month later showed little change, and he was continued with evaluation and laboratory monitoring every three months. This included complete blood count, comprehensive metabolic panel, lactate dehydrogenase, serum viscosity, serum protein electrophoresis, quantitative serum immunoglobulins, serum free light chains, and erythrocyte sedimentation rate (later replaced with CRP).

At nine months after diagnosis his hemoglobin was 12.2 g/dL, IgM was 4,010 mg/dL, and albumin remained low-normal at 3.5 g/dL. He had seen his internist for evaluation of mild gynecomastia, and that evaluation showed a low free testosterone level. Based on a case report of a patient with WM who had improvement of both his anemia and IgM after being treated for low testosterone [3], he opted to start a trial of topical testosterone at a dose titrated to maintain his free and total testosterone within the normal range. His hemoglobin increased over the next six months to 13.4 g/dL though there was no significant change in his IgM or albumin levels.

By 2½ years after diagnosis, his hemoglobin and albumin had decreased to 12.1 g/dL and 3.1 g/dL, respectively. He had developed mild bilateral dependent edema that was noticeable toward the end of the day, mild inguinal adenopathy, and some intermittent myalgias and paresthesias/hyperesthesias. He had no fever, chills, or other constitutional symptoms, and he continued to exercise regularly. Iron studies had shown repeated low iron saturations that had failed to respond to oral iron. There was no evidence of blood loss, and a gastroenterology evaluation including colonoscopy was normal with the exception of four 2 mm adenomatous polyps that were resected. Because WM is associated with elevated hepcidin which interferes with iron absorption and utilization, and because parenteral iron has been reported to reverse both the low iron saturation and accompanying anemia [4], he was started on a trial of intravenous iron.

A month after his initial iron infusion, his hemoglobin had risen to 13.1 g/dL with an appropriate reticulocyte response. However, over the next six months despite stabilization of his anemia, he began to develop increasing lower extremity edema, nocturia, and more frequent and severe myalgias. All these symptoms significantly worsened in the month prior to initiating chemotherapy. The lower extremity edema was now accompanied by periorbital edema, and it was the symptom having the greatest impact on his quality of life and daily activities. By the time he started chemotherapy, his albumin had dropped to 2.4 g/dL, IgM had risen to 5,550 mg/dL, and hemoglobin remained stable at 12.9 g/dL.

The edema was generalized, bilateral, and symmetric. There was no venous insufficiency, venous congestion, or venous prominence to suggest proximal venous obstruction. His inguinal adenopathy did not change during the time his edema increased, and there was no hepatosplenomegaly or other adenopathy on exam. Imaging was not done to look for obstructing adenopathy since it was felt that the likelihood was low, it would not have explained his periorbital edema, and its presence would not have changed treatment. History, physical exam, and laboratory studies did not reveal any evidence of cardiac, gastrointestinal, or hepatic disease, and he had no gastrointestinal symptoms that might have suggested the possibility of a protein-losing enteropathy. His serum protein electrophoresis also did not suggest protein-losing enteropathy. Despite his increasing edema, his exercise tolerance remained well above average for age, and there were no symptoms of cardiopulmonary disease such as might be seen with amyloid involvement. There were also no other signs of amyloid on physical exam such as weight loss, ecchymosis, tongue enlargement, or shoulder pad sign. Other than his hypoalbuminemia and edema, he had no signs or symptoms to suggest nephrotic syndrome or other renal disease. His serum creatinine and estimated glomerular filtration rate had remained normal and stable since the time of his diagnosis, as had his blood pressure. His only medications were levothyroxine, which he had been on since the time of his WM diagnosis, and the topical testosterone previously described. His thyroid function studies were consistently within the normal range as were his testosterone levels on a relatively low dose of topical testosterone. He was on no over-the-counter medications or dietary supplements other than a daily multivitamin and calcium tablet.

He was treated with the combination of bendamustine and rituximab every four weeks, with the first cycle limited to bendamustine alone to avoid an IgM flare. During the week following his first treatment, he monitored his fluid status, recording daily weights and fluid intake and output. He diuresed over 5 liters of fluid; his weight decreased by 6 kilograms; and his peripheral edema resolved. A 24-hour urine protein study was collected prior to his second cycle of chemotherapy and showed a total protein of 132 mg/24 h of which 20% was albumin. The urine M spike was 39 mg/24 h. Serum albumin was 3.3 g/dL, IgM was 2,680 mg/dL, and hemoglobin was 13.3 g/dL. Three months after completing four cycles of chemotherapy, his albumin was 4.7 g/dL, hemoglobin was 16.8 g/dL, and IgM was 317 mg/dL. He was asymptomatic; he was on no medications except levothyroxine; his inguinal adenopathy had resolved; and his exercise capacity had returned to levels that exceeded those prior to his WM diagnosis. At two years after treatment, he remains asymptomatic with no evidence of gastrointestinal or renal disease and no progression of his WM. IgM is 254 mg/dL, CRP is <3.0 mg/L, and albumin and hemoglobin are stable at 4.6 g/dL and 16.5 g/dL, respectively.

## 3. Discussion

Albumin is the most abundant plasma protein in humans. It helps to maintain colloid osmotic pressure, it binds numerous endogenous and exogenous compounds, and it provides the bulk of plasma antioxidant activity. Measured albumin concentration is the net result of liver synthesis, tissue catabolism, renal and gastrointestinal losses, and any non-steady state albumin space shifts that may occur in acute conditions such as sepsis or trauma. Elevated levels of inflammation-associated cytokines are associated with both decreased synthesis and increased catabolism of albumin, and hypoalbuminemia is seen in a myriad of acute and chronic conditions. In these conditions, its presence has frequently been identified as a predictor of increased morbidity and mortality [5–9].

Peripheral edema is also seen in a wide variety of conditions. Anything that alters the delicate balance of intravascular and interstitial hydrostatic and osmotic pressures, or increases capillary permeability, can result in edema formation. Although albumin helps to maintain colloid osmotic pressure, low levels of albumin alone do not typically result in edema. However, when combined with alterations of hydrostatic pressure, as occurs with increased intravascular volume and altered venous or lymphatic return, or with increased capillary permeability, as occurs with local or systemic inflammation, edema results [7, 10].

In WM, hypoalbuminemia has been reported as associated with both more severe disease and poorer prognosis, and it has also been identified as frequently present in symptomatic WM patients and in patients requiring treatment for their disease [11–13]. In one report of a cohort of symptomatic WM patients, albumin <3.5 g/dL was found in 70% of patients identified as “poor risk” (patients who survived <2 years after the initiation of therapy) and in 45% of the remaining patients [14]. But unlike the more common malignant monoclonal gammopathy multiple myeloma, albumin level has not historically been used for prognostic scoring or staging in WM [1, 15]. However, a recent study identified an albumin of 3.5 g/dL or less as one of four independent predictors of progression of asymptomatic WM to symptomatic WM. The other three were IgM 4,500 mg/dL or greater, bone marrow lymphoplasmacytic infiltration 70% or greater, and *β*2-microglobulin 4.0 mg/dL or greater. Using these four risk factors, the authors developed a validated risk model that categorizes patients as low, medium, or high risk based on median time to progression to symptomatic disease requiring treatment [16].

Despite these and other studies demonstrating a frequent association between hypoalbuminemia and WM, hypoalbuminemia is not typically identified as one of the manifestations of WM in review articles or in reference texts. It is also not identified as a factor to be considered in determining when to initiate treatment. This may be because gastrointestinal involvement, renal involvement, and nephrotic syndrome are rare in WM, and usually the result of amyloidosis, cryoglobulinemia, IgM or light chain deposition, or lymphoplasmacytic infiltration [17–20]. Yet most hypoalbuminemia seen in WM is not associated with these uncommon complications of WM, but is presumably due to the effects of elevated levels of inflammation-associated cytokines. This is consistent with what is seen in many other malignant and nonmalignant conditions in which acute and chronic inflammation are present [5–9].

This also appears to have been the case in our patient. The combination of his symptoms, physical findings, and laboratory results suggest that his WM resulted in a generalized inflammatory state that worsened as his disease progressed, and this inflammatory state did not resolve until his WM responded to treatment. This persistent inflammatory state likely resulted in a combination of hypoalbuminemia and increased capillary permeability that manifested as peripheral edema.

## 4. Conclusion

Hypoalbuminemia has been reported as common in patients with symptomatic WM, and it has recently been shown to be a risk factor for progression from asymptomatic to symptomatic disease requiring treatment. Despite this, it is not typically identified as a manifestation of WM in review articles or reference texts. As a result, clinicians seeing WM patients with hypoalbuminemia and edema might not attribute these findings to the disease but instead to other comorbid conditions commonly seen in the elderly population that is typical of WM. This case report and literature review illustrate that hypoalbuminemia is frequently associated with WM, it can improve when WM responds to treatment, and it should be included in lists of both the common and protean manifestations of the disease. Hypoalbuminemia may also become symptomatic, and it may be a reason to initiate treatment if symptoms affect quality of life.
